# Prevalence and molecular characterization of ticks and tick-borne pathogens of one-humped camels (*Camelus dromedarius*) in Nigeria

**DOI:** 10.1186/s13071-020-04272-2

**Published:** 2020-08-24

**Authors:** ThankGod E. Onyiche, Cristian Răileanu, Oliver Tauchmann, Susanne Fischer, Ana Vasić, Mandy Schäfer, Abdullahi A. Biu, Ndudim I. Ogo, Oriel Thekisoe, Cornelia Silaghi

**Affiliations:** 1 Institute of Infectology, Friedrich-Loeffler-Institut, Federal Research Institute for Animal Health, Südufer 10, 17493 Greifswald-Insel Riems, Germany; 2 Unit for Environmental Sciences and Management, North-West University, Potchefstroom Campus, Private Bag X6001, Potchefstroom, 2520 South Africa; 3 Department of Veterinary Parasitology and Entomology, University of Maiduguri, P. M. B. 1069, Maiduguri, 600230 Nigeria; 4 Parasitology Division, National Veterinary Research Institute, Vom, Plateau State Nigeria; 5 Department of Biology, University of Greifswald, Domstrasse 11, 17489 Greifswald, Germany

**Keywords:** Ticks, Tick-borne pathogens, Piroplasms, “*Candidatus* Anaplasma camelli”, Camels, Nigeria

## Abstract

**Background:**

Ticks are hematophagous arthropods responsible for maintenance and transmission of several pathogens of veterinary and medical importance. Current knowledge on species diversity and pathogens transmitted by ticks infesting camels in Nigeria is limited. Therefore, the aim of this study was to unravel the status of ticks and tick-borne pathogens of camels in Nigeria.

**Methods:**

Blood samples (*n* = 176) and adult ticks (*n* = 593) were collected from one-humped camels (*Camelus dromedarius*) of both sexes in three locations (Kano, Jigawa and Sokoto states) in north-western Nigeria and screened for the presence of *Rickettsia* spp., *Babesia* spp., *Anaplasma marginale*, *Anaplasma* spp. and *Coxiella*-like organisms using molecular techniques. All ticks were identified to species level using a combination of morphological and molecular methods.

**Results:**

Ticks comprised the three genera *Hyalomma*, *Amblyomma* and *Rhipicephalus*. *Hyalomma dromedarii* was the most frequently detected tick species (*n* = 465; 78.4%) while *Amblyomma variegatum* (*n* = 1; 0.2%) and *Rhipicephalus evertsi evertsi* (*n* = 1; 0.2%) were less frequent. Other tick species included *H. truncatum* (*n* = 87; 14.7%), *H. rufipes* (*n* = 19; 3.2%), *H. impeltatum* (*n* = 18; 3.0%) and *H. impressum* (*n* = 2; 0.3%). The minimum infection rates of tick-borne pathogens in 231 tick pools included *Rickettsia aeschlimannii* (*n* = 51; 8.6%); *Babesia* species, (*n* = 4; 0.7%) comprising of *B. occultans* (*n* = 2), *B. caballi* (*n* = 1) and *Babesia* sp. (*n* = 1); *Coxiella burnetii* (*n* = 17; 2.9%); and endosymbionts in ticks (*n* = 62; 10.5%). We detected DNA of “*Candidatus* Anaplasma camelli” in 40.3% of the blood samples of camels. Other tick-borne pathogens including *Anaplasma marginale* were not detected. Analysis of risk factors associated with both tick infestation and infection with *Anaplasma* spp. in the blood indicated that age and body condition scores of the camels were significant (*P* < 0.05) risk factors while gender was not.

**Conclusions:**

This study reports low to moderate prevalence rates of selected tick-borne pathogens associated with camels and their ticks in north-western Nigeria. The presence of zoonotic *R. aeschlimannii* emphasizes the need for a concerted tick control programme in Nigeria.
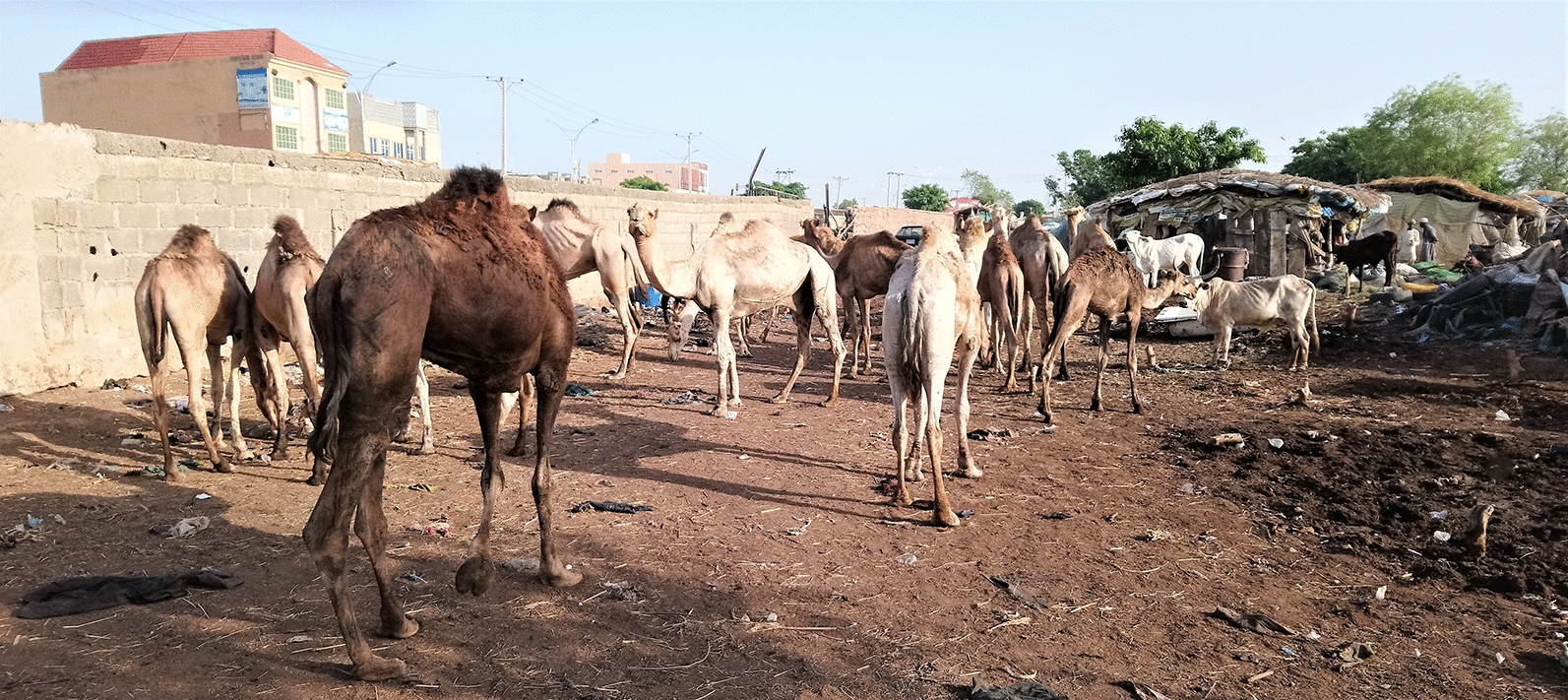

## Background

Ticks are responsible for substantial economic losses to farmers in livestock-keeping tropical regions of the world. Tick infestations cause wounds and inflammations due to tick bites, blood loss and potential diseases through transmission of pathogens [1]. The tick fauna infesting livestock in Africa is diverse with species belonging to the genera *Hyalomma*, *Rhipicephalus* and *Amblyomma*, having the highest impact on the productivity and health of these animals [2]. Tick-borne pathogens (TBPs) include viruses, bacteria, protozoans, and helminths afflicting humans’ and animals’ health worldwide [1]. Complex and dynamic interactions occur inside ticks with multiple microbes ranging from pathogens to endosymbionts [3]. The former is responsible for diseases, while the latter play a crucial role in maintaining fitness to the vector.

Tick-borne rickettsioses are caused by intracellular bacteria of the genus *Rickettsia*. Clinical manifestations include high fever, rash, myalgia, headache and lymphadenitis [4]. *Rickettsia africae* and *R. aeschlimannii* belong to the zoonotic spotted fever group (SFG) rickettsiae and have been reported from feeding hard ticks collected from livestock in Nigeria [5, 6].

Members of the genera *Anaplasma* and *Ehrlichia* (family *Anaplasmataceae*) can infect both animals and humans [7]. Limited studies have been conducted regarding the infection of camels with *Anaplasmataceae*. For example, *Anaplasma marginale* has been detected in camels using serological tests [8, 9]. However, other studies found no evidence of DNA of this bacterium [10, 11]. On the other hand, DNA of a novel species of *Anaplasma*, “*Candidatus* Anaplasma camelii” has been confirmed by sequencing in the blood of camels in various countries [12–14]. Infected animals may present clinical signs like anorexia, respiratory distress, edema of the sternum and xiphoid or even sudden death [13].

*Coxiella burnetii*, the causative agent of Q fever, is a zoonotic pathogen of vertebrates which is distributed worldwide [3]. Clinical manifestations are self-limiting febrile conditions in the majority of the cases and reproductive disorder in some animals [15]. Interestingly, strains of *Coxiella burnetii* have their origin from the diverse group of *Coxiella*-like endosymbionts, which are descendants of a *Coxiella*-like progenitor hosted by ticks [3].

Apicomplexan protozoans of the genus *Babesia* are transmitted by hard ticks [16]. Dromedaries are no exception to infection with *Babesia* although very few published reports exist so far [17, 18]. The pathogenicity differs according to the *Babesia* species. *Babesia caballi* causes severe clinical disease in equines characterized by fever, anemia, hemoglobinuria, and edema in some cases [19], while *B. occultans* is of lower pathogenicity in animals as previously reported in cattle with no visible clinical signs [20]. In camels, reported clinical signs of babesiosis includes anemia, fever, icterus, hemoglobinuria, and gastro-intestinal stasis [21].

Current estimates in Nigeria on the one-humped camel (*Camelus dromedaries*) population are at about 283,395 heads [22]. Pastoralists primarily keep these animals for transportation and as source of meat. The carcass yield from camels are higher under cheap management system. Recent estimates show that the consumption of camel meat in Nigeria has increased substantially due to its nutritional value and for health reasons [23]. Camel meat has relatively less fat compared to cattle and sheep and is acclaimed to cure diseases like hypertension, hyperacidity, and cardiovascular disease [24]. On the other hand, researchers in Nigeria consider the dromedary camel as a ‘foreign animal’ and this has led to research apathy on this animal species in recent past [25]. As desertification continues to encroach into sub-Saharan Africa, renewed interest is also gradually building up in northern Nigeria, as the camel is resilient to the arid land conditions and seems certainly the best option to mitigate the effects of environmental conditions on livestock production among the pastoralist in northern Nigeria [26]. In Nigeria, dromedary camels are raised in semi-arid conditions grazing on poor pastures for most of the year where they are exposed to a wide variety of vectors including ticks. This shows the need to ascertain this source of potential disease. In order to be better prepared to raise this animal species successfully without the debilitating effects of ticks and tick-borne disease on their health and productivity, this study was carried out to assess (i) the species diversity of ticks on camels, (ii) the occurrence of selected tick-borne pathogens in ticks collected and blood taken from camels and (iii) the risk factors associated with infection of camels with tick-borne pathogens in Nigeria.

## Methods

### Study area

**Fig. 1:**
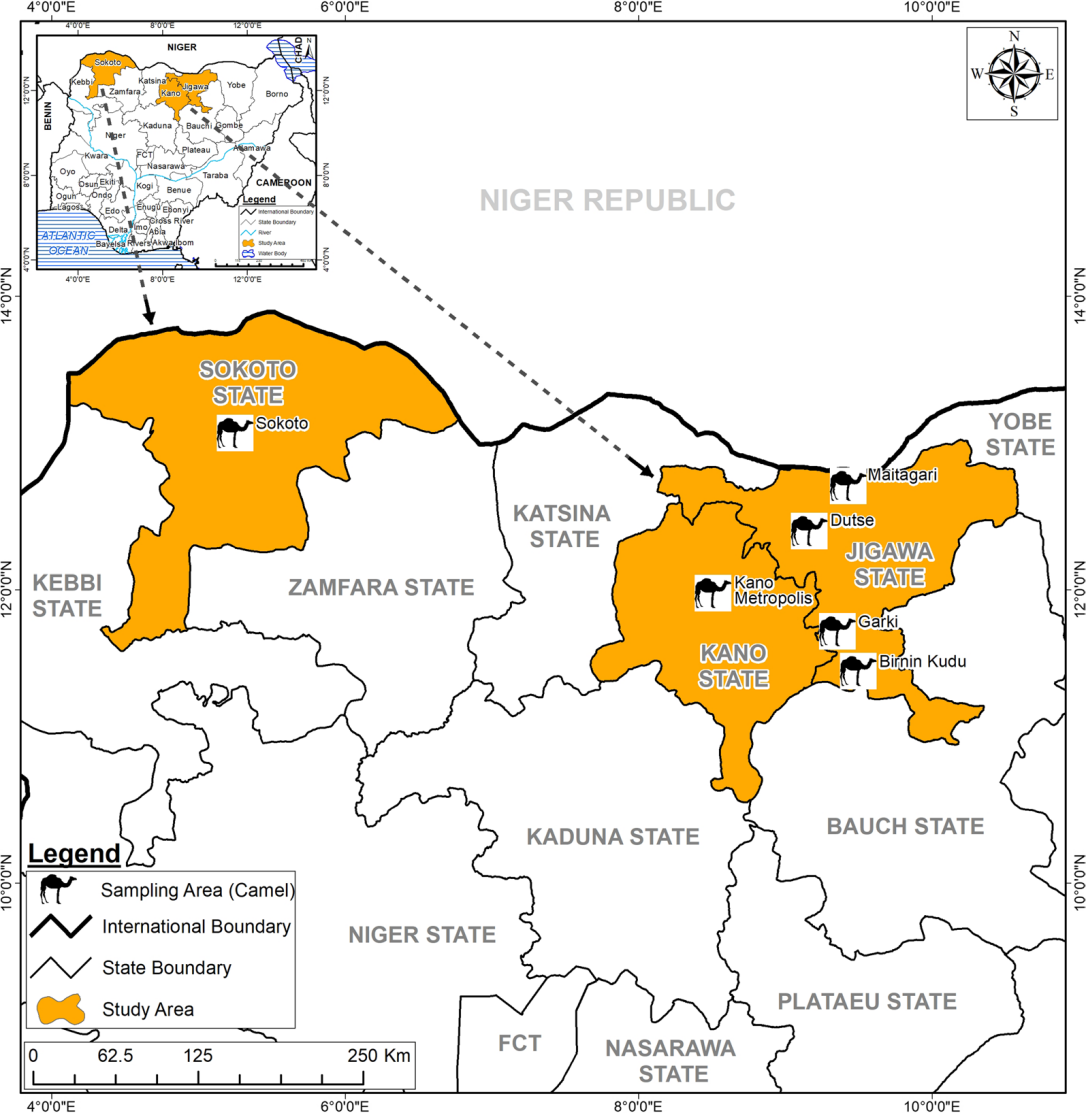
Map of Nigeria with insert of the northwestern region showing the study areas where samples were collected (Maps were created using ArcGIS version 10.6 by ESRI, Redlands, CA, USA)

The North-West region is a semi-arid zone and the largest region in Nigeria with a combined human population of 35,786,944 [27]. This region has a savannah type of vegetation favorable to camel husbandry because they are easily predisposed to foot rot associated with wetland and this hence the concentration of camels at this region [28]. The temperature ranges from 18 °C to 45 °C with a mean temperature of 27 °C. There is a single rainy season from May to October with mean annual rainfall of 508–1016 mm. Three states (Sokoto, Jigawa and Kano) were selected for sampling (Fig. 1).

### Study design and sampling locations

A cross sectional study was carried out from September to November 2017. Additional samples were collected in November 2018. Non-probability sampling, combining both convenient and snowball sampling techniques were employed. Blood and tick samples were collected from several sampling points across the three study areas comprising of abattoirs, livestock markets and herders/pastoralists. All samples from Kano (*n* = 92) were collected from the Kano metropolitan abattoir (12.0123540N, 8.520795E) located in the city of Kano. For Sokoto state, all samples (*n* = 55) were collected from herders/pastoralists at several locations within the state. The geographical coordinates for the state of Sokoto are 12.1358N, 4.8654E. Finally, the livestock market located in Maigatari local government of Jigawa state (12.8125483N, 9.444303E) as well as adjoining local villages within this area were used for sampling in Jigawa state (*n* = 29). Sampled animals from all study areas were raised under the traditional nomadic (extensive) management system typical of camel husbandry in Africa with little access to veterinary care. Information such as age (< 5 years/> 5 years), sex (male/female) and presence/absence of ticks were collected for each animal to assess possible risk factors associated with tick-borne pathogen infection. Body condition score were classified into any of the three classes (poor, moderate and good) based on the fat storage at the back and flank region using visual inspection. All samples were collected from animals that were apparently healthy without any clinical signs of infection after seeking the owner’s consent and approval.

### Blood and tick sample collection

About 5 ml of whole blood were collected from the jugular vein and in some cases from the lateral abdominal vein of clinically healthy animals and in the case of slaughtered animals, from severed jugular blood vessels. All collected blood samples were transferred into labelled EDTA coated tubes and transported to the laboratory on ice packs within 4 h. In the laboratory, 125 µl of blood was dispensed on the marked spot within the Classic FTA card (Whatman ® GE Healthcare, Buckinghamshire, UK). All cards were labeled, air dried and stored at room temperature for further analysis. The skin of the camels covering known predilection sites for ticks including the perineum region, abdomen and thigh, ear, neck, and dewlap were carefully examined for the presence of ticks. Ticks were collected using tweezers into labelled tubes plugged with cotton. Ticks from each animal were kept in separate tubes. The labelled tubes contained information on the identity of the animal including their location.

### Morphological identification of ticks

All tick samples collected from infested animals were identified to species level based on standard keys using a stereomicroscope (Olympus®, Tokyo, Japan) separately by two of the co-authors [29]. Specimens were separated based on species, life stage and sex. All tick specimens were preserved in 70% ethanol and kept at 4 °C after identification.

### Washing and homogenization of ticks

Individual ticks were washed twice with double distilled water after the removal of ethanol in individual Eppendorf tubes as described by Silaghi et al. [30]. A 5 mm sterile stainless-steel bead and 100 µl of sterile PBS were added to each tube. Ticks were homogenized using Tissue Lyser II (Qiagen, Hilden, Germany) for 60 s twice with 30 s break in between at an oscillation frequency of 30 Hz. After centrifugation at 2500× *rpm* for 3 min, the supernatant was removed.

### Pooling of supernatant and extraction of genomic DNA from tick homogenates and FTA cards

Prior to extraction, the supernatants from ticks of the same species and the same animal were pooled with a maximum of 5 ticks per pool. A maximum of 80 µl of the homogenate (supernatant) was used for DNA extraction (individual tick contributing a maximum of 16 µl). For partially-fed ticks, supernatants were either pooled or used individually (engorged ticks were used individually). Extraction of DNA from FTA cards (blood) was performed from approximately a 6 mm punch from the dried blood spot on the card. The spot was carefully excised into a sterile 2 ml labelled Eppendorf tube containing one 4 mm sterile stainless-steel bead. The samples were then lysed using Tissue Lyser II (Qiagen) for 60 s twice. Isolation of genomic DNA was carried out with QIAamp DNA Mini Kit (Qiagen) according to manufacturer’s instruction. Genomic DNA was stored at − 20 °C until use.

### Tick species identification using PCR

For the molecular identification of tick species, three different genes (*12S* rRNA, *16S* rRNA and *cox*1) were targeted using primer pairs shown in Table 1. Genetic identification of ticks was carried out using DNAs extracted from a single tick representative of each species. The reaction was performed in total volume of 25 μl using the GoTaq® G2 Flexi DNA Polymerase Kit (Promega, Madison, WI, USA). The PCR mix consisted of 5 μl GoTaq® 5× Flexi Buffer (green), 3 μl 25 mM MgCl2 solution, 0.5 μl 10 mM dNTPs, 1 μl of each primer (both forward and reverse) (10 µM), 0.1 μl of GoTaq® DNA Polymerase (5 u/μl), 9.4 µl nuclease-free water (NFW) and 5 µl template DNA. A thermal cycler C1000 (Bio-Rad, Munich, Germany) was used for amplification and the cycling conditions are provided in Table 1.

**Table 1:** Primer sets used for DNA amplification and sequencing of ticks and tick-borne pathogens in ticks and camels from north-western Nigeria

Target	Method	Gene target	Primer sequence (5′-3′)	Product size (bp)	Positive control (DNA) from ticks	Reference
Tick identification	PCR	*12S* rRNA	T1B: AAACTAGGATTAGATACCCT	360	*Hyalomma dromedarii*	[35]
			T2A: AATGAGAGCGACGGGCGATGT			
Tick identification	PCR	*16S* rRNA	16S + 1: CTGCTCAATGATTTTTTAAATTGCTGTGG	456	*H. dromedarii*	[36]
			16S − 1: CCGGTCTGAACTCAGATCAAGTA			
Tick identification	PCR	*cox*1	Cox1F: GGAACAATATATTTAATTTTTGG	360	*H. dromedarii*	[37]
			Cox1R: ATCTATCCCTACTGTAAATATATG			
*Rickettsia* spp.	PCR	*gltA*	Rsfg877: GGGGGCCTGCTCACGGCGG	381	*Rickettsia helvetica*	[38]
			Rfsg1258: ATTGCAAAAAGTACAGTGAACA			
*Rickettsia* spp.	PCR	*ompA*	Rr190.70p: ATGGCGAATATTTCTCCAAAA	631	*R. helvetica*	[39]
			Rr190.701n: GTTCCGTTAATGGCAGCATCT			
*Rickettsia* spp.	PCR	*ompB*	120–2788: AAACAATAATCAAGGTACTGT	765	*R. helvetica*	[40]
			120–3599: TACTTCCGGTTACAGCAAAGT			
*Babesia/Theileria*	PCR	*18S* rRNA	BJ1: GTCTTGTAATTGGAATGATGG	411–452	*Babesia* spp.	[41]
			BN2: TAGTTTATGGTTAGGACTACG			
			BabsppF1: GTTTCTGMCCCATCAGCTTGAC	422–440		[42]
			BabsppR: CAAGACAAAAGTCTGCTTGAAAC			
*Anaplasma marginale*	qPCR	*msp1ß*	AM-forward: TTGGCAAGGCAGCAGCTT	95	*Anaplasma marginale*	[31]
			AM-reverse: TTCCGCGAGCATGTGCAT			
			AM-probe: FAM TCGGTCTAACATCTCCAGGCTTTCAT BHQ1			
*Anaplasma/Ehrlichia*	PCR	*16S* rRNA	EHR16SD: GGTACCYACAGAAGAAGTCC	345	*A. marginale*	[43]
			EHR16SR: TAGCACTCATCGTTTACAGC			
*Anaplasma* spp.	Semi-nested PCR	*16S* rRNA	fD1: AGAGTTTGATCCTGGCTCAG	760	*A. marginale*	[44]
			EHR16SR: TAGCACTCATCGTTTACAGC			
			fD1: AGAGTTTGATCCTGGCTCAG	426		[45]
			GA1UR: GAGTTTGCCGGGACTTCTTCT			
*Coxiella*-like organisms	Semi-nested PCR	*16S* rDNA	Cox16SF1: CGTAGGAATCTACCTTRTAGWGG	1321–1416	*Coxiella burnetii*	[46]
			Cox16SR2: GCCTACCCGCTTCTGGTACAATT			
			Cox16SF2: TGAGAACTAGCTGTTGGRRAGT	624–625		
			Cox16SR2: GCCTACCCGCTTCTGGTACAATT			

### Molecular detection of pathogens using PCR

For pathogen detection, PCRs were used for amplification of DNA of *Rickettsia* spp., *Anaplasma/Ehrlichia* spp., *A. marginale*, *C. burnetii* and *Babesia/Theileria* spp. from tick DNA, while *A. marginale*, “*Ca.* A. camelii” and *Babesia/Theileria* spp. were screened from blood DNA. All reactions were performed in total volume of 25 μl using the GoTaq® G2 Flexi DNA Polymerase Kit (Promega) The PCR mix contained 5 μl GoTaq® 5× Flexi Buffer (green), 3 μl 25 mM MgCl_2_ solution, 0.5 μl 10 mM dNTPs, 400 nM of each primer (forward and reverse), 0.1 μl of GoTaq® DNA Polymerase (5 u/µl), 9.4 µl NFW and 5 µl of template DNA. Every reaction set had a positive and negative control (molecular grade water). Table 1 summarizes the PCR cycling conditions.

### Gel electrophoresis and sequencing

Agarose gel electrophoresis at a concentration of 1.5% was used for the separation of PCR products with 2 μl GelRed™ (1×; equivalent to 1 µl/10 ml) (Biotium Fremont, CA, USA). Bands were visualized using a ChemiDoc™ MP imaging system (Bio-Rad). Amplicons were purified with NucleoSEQ® columns (Macherey-Nagel, Düren, Germany), according to the manufacturer’s instructions, and then sequenced in one direction using an ABI PRISM® 3130 sequencer (Applied Biosystem, California, USA) at the Institute of Diagnostic Virology, Friedrich Loeffler Institute, Germany. The nucleotide sequences were viewed and edited using Geneious 9.1 software (Biomatters, Auckland, New Zealand) and analyzed against sequences deposited in GenBank using BLASTn (National Centre for Biotechnology Information; www.blast.ncbi.nlm.nih.gov/Blast) for high similarity sequences.

### Real-time PCR for the amplification of *A. marginale*

The *msp1ß* gene of *A. marginale* was targeted in DNA samples from ticks and camel blood using species-specific primers and probe (Table 1) as previously described [31, 32]. The PCR was carried out using a CFX-96 Real-Time System (Bio-Rad) with the cycling conditions described in Table 1. PCR amplification was carried out using iTaq™ Universal Probes Supermix (Bio-Rad) in a total volume of 25 µl comprising of 200 nM of forward and reverse primers, 100 nM of probe (Table 1), 12.5 µl (2×) iTaq™ Supermix, 0.9 µl RNase free water and 10 µl template DNA. Each reaction run included a positive and negative control.

### Statistical analysis

For pooled tick samples, the prevalence was estimated using the minimum infection rate (MIR). MIR assumes that only one tick is infected in a positive pool [33]. Results are also presented with a 95% confidence interval (CI: lower and upper) for the infection rates and MIR for the detected pathogens. MIR was expressed in simple percentages (only one tick was considered as positive, in a pool of adult ticks). The calculation was carried out thus MIR = (P/N) × 100%, where P is the number of positive pools, N is the total number of ticks tested. Chi-square test was used to test for statistical significance between the various risk factors. The odds ratio was used to test the association/likelihood of the presence of ticks and infection with *Anaplasma* spp. The level of significance was set as *P* < 0.05. Statistical significance was carried out using GraphPad Prism version 5.0 (GraphPad Software, La Jolla California USA; www.graphpad.com).

### Phylogenetic analysis

The nucleotide sequences were viewed and edited using Geneious 11.1.5 and analyzed against references in GenBank using BLASTn (www.blast.ncbi.nlm.nih.gov/Blast) for high similarity sequences to confirm identity for the ticks as well as for pathogens. Sequences were added to alignment explorer in MEGA 7 and aligned with ClustalW [33]. Reference sequences were also added to the aligned datasets. Model test was run in MEGA 7 prior to the tree construction in order to select the suitable model. The phylogenetic tree was constructed using the Maximum Likelihood method based on the Kimura 2-parameter model [34] with 1000 replicates. Median joining network was constructed using PopART (http://popart.otago.ac.na) to examine the haplotype distribution and relationships.

## Results

### Morphological and molecular identification of tick species

Of the 176 camels examined, 92 (52.3%) were infested with ticks from a total collection of 593. All ticks collected were identified as adult with no immature stages comprising of 440 (74.2%) males and 153 (25.8%) females. The largest number of ticks was collected from Kano (396; 66.8%) followed by Jigawa (145; 24.5%) and Sokoto (52; 8.8%) state (Table 2).

**Table 2:** Demography of adult tick species infesting 176 camels in north-western Nigeria

Parameter/Tick species	Total number (%)	Study locations					
		Kano (*n* = 396)		Jigawa (*n* = 145)		Sokoto (*n* = 52)	
		Male	Female	Male	Female	Male	Female
*Hyalomma dromedarii*	465 (78.41)	254	75	87	27	12	10
*H. truncatum*	87 (14.67)	22	18	15	6	22	4
*H. rufipes*	19 (3.20)	3	5	7	1	3	0
*H. impeltatum*	18 (3.03)	12	4	1	0	1	0
*H. impressum*	2 (0.34)	0	2	0	0	0	0
*Amblyomma variegatum*	1 (0.17)	1	0	0	0	0	0
*Rhipicephalus evertsi evertsi*	1 (0.17)	0	0	0	1	0	0
Total	593 (100.0)	292 (49.2)	104 (17.5)	110 (18.5)	35 (5.9)	38 (6.4)	14 (2.4)

Altogether, 7 species of ticks were identified: *Hyalomma dromedarii* (*n* = 465; 78.4%), *H. truncatum* (*n* = 87; 14.7%), *H. rufipes* (*n* = 19; 3.2%), *H. impressum* (*n* = 2; 0.3%), *H. impeltatum* (*n* = 18; 3.0%), *Amblyomma variegatum* (*n* = 1; 0.2%) and *Rhipicephalus evertsi evertsi* (*n* = 1; 0.2%) (Table 2). Three tick species (*H. dromedarii*, *H. truncatum* and *H. rufipes*) were found in all three locations while *H. impeltatum* was found only in Kano and Sokoto. *Amblyomma variegatum* was collected in Kano state and *R. e. evertsi* in Jigawa state only and lastly, *H. impressum* in Kano state (Table 2). To confirm the identity of these species of ticks, molecular identification was carried out. A BLASTn query of the obtained sequences for the *16S* rRNA gene revealed a high identity match ranging from 98.9% to 100% for all tick species except for *H. rufipes*. Due to ambiguity of the *16S* rRNA gene for *H. rufipes*, the *12S* rRNA gene was amplified and a BLASTn query of the obtained sequences still could not clear this ambiguity. Lastly, we amplified the *cox*1 gene followed by sequencing. The sequence analysis gave 99.8% homology with *H. rufipes* (GenBank: KX000641.1). The newly generated sequences were deposited in the GenBank database under the accession numbers MN394427-MN39444 (*16S* rRNA gene), MN394457-MN394461 (*12S* rRNA gene) and MN601291-MN601294 (*cox*1).

### Risk factors associated with tick infestations of camels

Tick infestation rates were slightly higher in male camels across the three different locations with 65.6% (40 /61) in Kano, 63.2% (12/19) in Jigawa and 27.8% (10/36) in Sokoto compared with female camels with 64.5% (20/31), 60.0% (6/10) and 21.1% (4/19), respectively. No significant difference was observed between sexes across the study locations (*P* > 0.05) (Table 3). The infestation rate was significantly higher in camels > 5 years-old compared with those < 5 years-old and differed significantly (*P* < 0.05) (Table 3). The odds of infestation with ticks were higher in camels > 5 years-old from Kano (OR: 1.44, 95% CI: 0.49–4.18) compared with Jigawa (OR: 0.76, 95% CI: 0.15–4.30) and Sokoto states (OR: 0.49, 95% CI: 0.14–1.73) (Table 3). Finally, camels with a good body condition score were significantly less infested with ticks across the three study locations compared with those with either poor or moderate body condition score (*P* < 0.05) (Table 3).

**Table 3:** Risk factors associated with infestation of 176 camels with ticks in north-western Nigeria

Risk factor	*n*	Total no. infested with ticks (%)	Study location								
			Kano			Jigawa			Sokoto		
			No. examined	No. infested (%)	Odds ratio (95% CI)	No. examined	No. infested (%)	Odds ratio (95% CI)	No. examined	No. infested (%)	Odds ratio (95% CI)
Sex											
Male	116	62 (53.5)	61	40 (65.6)	Ref	19	12 (63.2)	Ref	36	10 (27.8)	Ref
Female	60	30 (50.0)	31	20 (64.5)	1.05 (0.42–2.59)	10	6 (60.0)	1.14 (0.24–5.50)	19	4 (21.1)	1.44 (0.38–5.41)
Total	176	92 (52.3)	92	60 (65.2)		29	18 (62.2)		55	14 (25.5)	
Age											
< 5 years	66	27 (40.9)	21	15 (71.4)	Ref	7	4 (57.1)	Ref	38	8 (21.1)	Ref
> 5 years	110	65 (64.5)	71	45 (47.9)	1.44 (0.49–4.18)	22	14 (63.6)	0.76 (0.15–4.30)	17	6 (42.9)	0.49 (0.14–1.73)
Total	176	92 (52.3)	92	60 (65.2)		29	18 (62.1)		55	14 (25.5)	
Body condition											
Poor	10	7 (70.0)	7	4 (57.1)	Ref	3	2 (66.7)	Ref	3	1 (33.3)	
Moderate	158	81 (51.3)	81	55 (67.9)	0.63 (0.13–3.03)	25	16 (64.0)	0.63 (0.05–8.44)	47	10 (21.3)	1.85 (0.15–22.55)
Good	8	4 (50.0)	4	1 (25.0)	4.00 (0.27–60.37)	1	0 (0)	1.22 (0.0915.12)	5	3 (60.0)	0.33 (0.02–6.66)
Total	176	92 (52.3)	92	60		29	18 (62.1)		55	14 (25.5)	

*Abbreviation*: n, total number; Ref, reference value

### Molecular detection of tick-borne pathogens in ticks

#### *Rickettsia* spp.

Altogether, 67 out of 231 tick pools (comprising of 593 ticks) produced bands of the correct length in the *gltA* PCR and all were sequenced. Out of those, 51 resulted in good quality sequences and could be evaluated as *Rickettsia* spp. Therefore, the minimum infection rate (MIR) for tick pools for *Rickettsia* spp. was 8.6%. Across the study locations, 31 pools were positive (MIR 7.8%) in Kano state, 14 pools (MIR 9.7%) in Jigawa state and 6 pools (MIR 11.5%) in Sokoto state (Table 4). No significant difference (*P* > 0.05) was observed between the different study locations.

**Table 4:** Minimum infection rates of tick-borne pathogens detected in tick pools from different study locations in north-western Nigeria

Study location	Total no. of ticks tested (*n*)	Minimum infection rate % (number of positives) [95% CI]		
		*Rickettsia* spp.(*n*)	*Babesia* spp. (*n*)	*Coxiella burnetii* (*n*)
Kano	396 (150)	7.8 (31) [5.4–10.9]	0.8 (3) [0.2–2.2]	3.0 (12) [1.6–5.2]
Jigawa	145 (57)	9.7 (14) [5.4–15.7]	0.7 (1) [0.0–3.8]	2.1 (3) [0.4–5.9]
Sokoto	52 (24)	11.5 (6) [4.4–23.4]	0 (0)	3.8 (2) [0.5–13.2]
Total	593 (231)	8.6 (51) [6.5–11.2]	0.7 (4) [0.2–1.7]	2.9 (17) [1.7–4.6]

*Abbreviation*: n, number of pools

*Rickettsia* spp. was detected in four different tick species with *H. rufipes* having the highest MIR (36.8%) and *H. impeltatum* with the lowest MIR (5.6%). Others include *H. truncatum* (16.1%) and *H. dromedarii* (6.2%) (Table 5).

**Table 5:** Minimum infection rate of tick-borne pathogens in tick pools in relation to tick species collected from camels in north-western Nigeria

Study location	Total no. of ticks tested (*n*)	Minimum infection rate (no. of positives) [95% CI]		
		*Rickettsia* spp. (*n*)	*Babesia* spp. (*n*)	*Coxiella burnetii* (*n*)
*Hyalomma dromedarii*	465 (154)	6.2 (29) [4.2–8.8]	0.6 (3) [0.1–1.9]	3.4 (16) [2.0–5.5]
*H. truncatum*	87 (46)	16.1 (14) [9.1–25.5]	0 (0)	1.1 (1) [0.0–6.2]
*H. rufipes*	19 (16)	36.8 (7) [16.3–61.6]	0 (0)	0 (0)
*H. impeltatum*	18 (11)	5.6 (1) [0.1–27.3]	5.6 (1) [0.1–27.3]	0 (0)
*H. impressum*	2 (2)	0 (0)	0 (0)	0 (0)
*Amblyomma variegatum*	1 (1)	0 (0)	0 (0)	0 (0)
*Rhipicephalus evertsi evertsi*	1 (1)	0 (0)	0 (0)	0 (0)
Total	593(231)	8.6 (51) [6.5–11.2]	0.7 (4) [0.2–1.7]	2.9 (17) [1.7–4.6]

*Abbreviation*: n, number of pools

Following a BLASTn query on the NCBI database, 45 of these sequences showed 100% identity with *R. aeschlimannii* (GenBank: MH267736.1) and 6 showed high similarity scores ranging between 98.7–99.7% (GenBank: MH267736.1). BLASTn analysis of one of the sequences obtained from the *gltA* gene of *Rickettsia* spp., gave 100% homology with *C. burnetii* (GenBank: CP035112.1).

To further confirm the genotypes of *Rickettsia* spp., *ompA* and *ompB* genes were partially amplified. We tested all *gltA-*positive samples with good quality sequences (*n* = 51) for both *ompA* and *ompB*. For *ompA* and *ompB*, 39 and 43 tick pools, respectively were positive, from which 13 and 16 amplicons were selected for sequencing and gave good quality sequences. BLASTn analysis of these sequences obtained for both genes showed 99.9–100% similarity with *R. aeschlimannii* on GenBank. All newly generated sequences were deposited in the GenBank database under the accession numbers MN601304-MN601344 (*gltA*), MT126809-MT126818 (*omp*A) and MN601295-MN601303 (*omp*B).

#### *Anaplasma*/*Ehrlichia* spp.

The PCR targeting the *16S* rRNA gene of *Anaplasma/Ehrlichia* spp. was positive in 62 out of 231 tick pools with a MIR of 10.5%. Based on location, the MIR in Sokoto state was 15.4% followed by Kano state with 10.1% while Jigawa state had the lowest MIR of 9.7%. No significant difference (*P* > 0.05) was observed across the different sampling locations (Table 4).

Five different tick species were positive: *H. dromedarii* (MIR, 8.0%), *H. truncatum* (MIR, 16.1%), *H. rufipes* (MIR, 36.8%), *H. impeltatum* (MIR, 11.1%) and *H. impressum* (MIR, 100.0%) (Table 5).

BLASTn analysis of sequences obtained from positive samples of the *Anaplasma/Ehrlichia* PCR had sizes of 240 bp with 98–100% similarity to *Peptoniphilus* spp. (GenBank: LC145547.1), “*Candidatus* Midichloria mitochondrii” (GenBank: KU559921.11) and a *Rickettsiales* bacterium (GenBank: DQ379964.1).

#### *Anaplasma marginale*

*Anaplasma marginale* DNA was not detected in DNA from ticks.

#### *Coxiella burnetii*

The DNA of *C. burnetii* was detected in 17 out of 231 tick pools with a MIR of 2.9%. The MIR for the states of Sokoto, Kano and Jigawa was 3.8%, 3.0% and 2.1%, respectively (Table 4). No significant difference (*P* > 0.05) was observed across the different study locations. Most *C. burnetii* positive tick pools were *H. dromedarii* (MIR 3.4%) and *H. truncatum* (MIR 1.1%) (Table 5). Only 1 out of 87 (1.1%) *H. truncatum* ticks in the pools were positive to *C. burnetti* (Table 5). Sequences had similarity scores ranging between 99.2–100% to *C. burnetii* (GenBank: CP035112.1). The newly generated sequences were deposited in the GenBank database under the accession numbers MN396571-MN396578.

### *Babesia* spp.

The MIR for *Babesia* spp. was 0.7% (4/593) across the study locations with 3 positives in Kano state (MIR 0.8%) and one in Jigawa state (MIR 0.7%) (Table 4). No significant difference (*P* > 0.05) was observed across the different sampling locations. Three out of the four positive pools were detected in *H. dromedarii* (MIR 0.6%) and one was detected in *H. impeltatum* (MIR 5.6%) (Table 5).

BLASTn analysis showed that 2 sequences showed 100% identity to *B. occultans* (GenBank: MG920540.1), 1 with 100% identity to *B. caballi* (GenBank: MG052892.1) and 1 showed 98.5% similarity to *Babesia* spp. (GenBank: KC249945.1). A further attempt to characterize the undifferentiated species of *Babesia* using a different primer pair [41] showed 100% homology with *Babesia* spp. (GenBank: KC249946.1). The newly generated sequences were deposited in the GenBank database under the accession numbers MN394378-MN394381.

### Co-detection of tick-borne pathogens in ticks

A low co-detection rate was observed in the study with all co-detections occurring in Kano state only. Co-detection was observed for *Rickettsia* spp. + *Babesia* spp. in one tick pool as well as for *Rickettsia* spp. + *C. burnetii* in another tick pool.

### Molecular detection of tick-borne pathogens in the blood of camels

#### *“Candidatus Anaplasma camelii”*

The overall prevalence of “*Ca*. A. camelii” from the three study locations was 40.3% (71/176). Kano state had the highest prevalence of 59.8% (55/92), followed by Jigawa with 37.9% (11/29) and Sokoto state with 9.1% (5/55) (Table 6).

**Table 6:** Risk factors associated with infection with “*Candidatus* Anaplasma camelli” in blood collected from camels in north-western Nigeria

Parameter	*n*	Total no. infected (%)	Study location					
			Kano		Jigawa		Sokoto	
			No. examined	No. infected (%)	No. examined	No. infected (%)	No. examined	No. infected (%)
Sex								
Male	116	41 (35.34)	61	31 (50.82)	19	7 (36.84)	36	3 (8.33)
Female	60	30 (50.00)	31	24 (77.41)	10	4 (40.0)	19	2 (10.53)
Total	176	71 (40.34)	92	55 (59.78)	29	11 (37.93)	55	5 (9.09)
Age								
< 5 years	66	20 (30.30)	21	15 (71.43)	7	2 (28.57)	38	3 (7.89)
> 5 years	110	51 (46.36)	71	40 (56.34)	22	9 (40.91)	17	2 (11.76)
Total	176	71 (40.34)	92	55 (59.78)	29	11 (37.93)	55	5 (9.09)
Body condition								
Poor	10	4 (40.00)	7	3 (42.86)	3	1 (33.33)	3	0 (0)
Moderate	158	66 (41.77)	81	52 (64.19)	25	10 (40.0)	47	4 (8.51)
Good	8	1 (12.50)	4	0 (0)	1	0 (0)	5	1 (20.0)
Total	176	71 (40.34)	92	55 (59.78)	29	11 (37.93)	55	5 (9.09)
Presence of ticks								
Yes	92	42 (45.65)	60	35 (58.33)	18	6 (33.33)	14	1 (7.14)
No	84	29 (34.52)	32	20 (62.5)	11	5 (45.45)	41	4 (9.76)
Total	176	71 (40.34)	92	55 (59.78)	29	11 (37.93)	55	5 (9.09)

*Abbreviation*: n, total number

GenBank analysis of representative sequences (*n* = 15) selected from all the study locations with a product size of 345 bp showed 99.6–100% similarity to *16S* rDNA of *Anaplasma platys* (GenBank: MH762081.1) and “*Ca*. A. camelii” (GenBank: KF843827.1). In the attempt to differentiate these two species, semi-nested PCR targeting the *16S* rRNA gene of *Anaplasma* spp. was used, generating a PCR product of 426 bp. BLASTn analysis of the sequences yielded “*Ca*. A. camelii” (GenBank: KF843825.1) with the highest identity score of 100% (GenBank: KF843825.1). The newly generated sequences were deposited in the GenBank database under the accession numbers MN396629-MN396638.

#### *Babesia* spp. and *Anaplasma marginale*

DNA of neither pathogen was amplified in the blood of camels.

### Risk factors associated with “*Candidatus* A. camelii” infection in blood of camels

A higher number of female camels were infected as compared to males, although no significant difference (*P* > 0.05) was observed (Table 6). Furthermore, the prevalence was higher in camels > 5 years-old across the three study areas compared with those < 5 years-old old. A significant difference was observed between age groups (*P* < 0.05) (Table 6).

Camels with poor or moderate body condition had higher infection rates with “*Ca*. A. camelii” compared to those with a good body condition with a significant difference (*P* < 0.05). Only one camel (20.0%, 1/5) with a good body condition score was infected in Sokoto state (Table 6). Finally, camels infested with ticks were two times more likely to be infected with “*Ca*. A. camelii” compared with those without ticks (OR: 1.59, 95% CI: 0.9–2.9).

### Phylogenetic and haplotype analysis of “*Ca*. A. camelii”

**Fig. 2:**
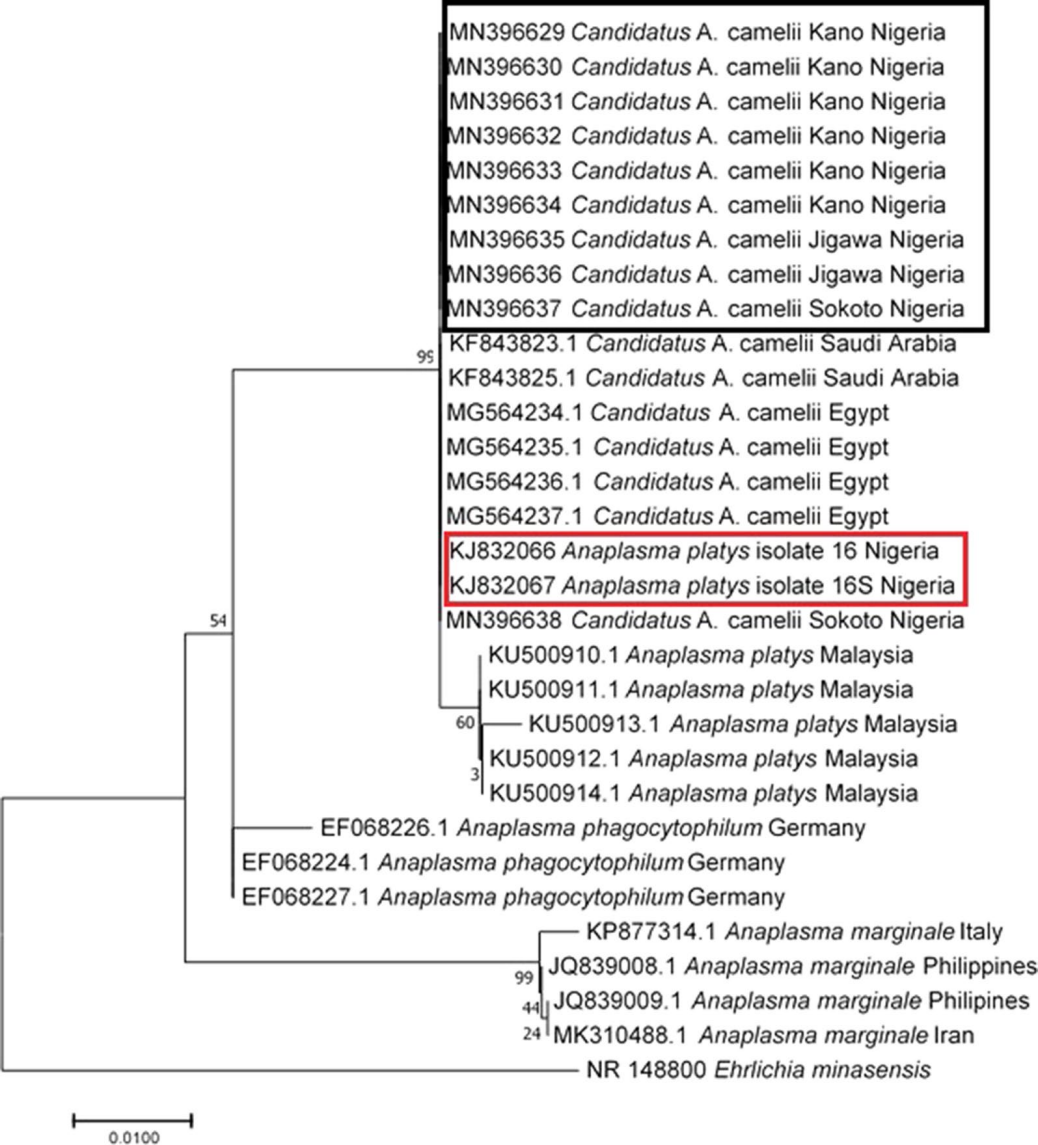
Phylogenetic tree based on *16S* rDNA sequences of “*Candidatus* A. camelii” isolates identified in this study (indicated in the black box) and *Anaplasma platys* sequences from a previous study from Sokoto, Nigeria, retrieved from GenBank (indicated in the red box). The Maximum Likelihood method based on the Kimura 2-parameter model was used to construct the tree at 1000 replicates using MEGA 7. *Ehrlichia minasensis* was used as the outgroup

“*Candidatus* A. camelii” nucleotide sequences from this study clustered together with all other “*Ca*. A. camelii” sequences from Saudi Arabia (GenBank: KF843823-KF843825) and Egypt (GenBank: MG564235-MG564237) (Fig. 2). In addition, *A. platys* sequences from a previous study in Nigeria clustered with the sequences from this study.

**Fig. 3:**
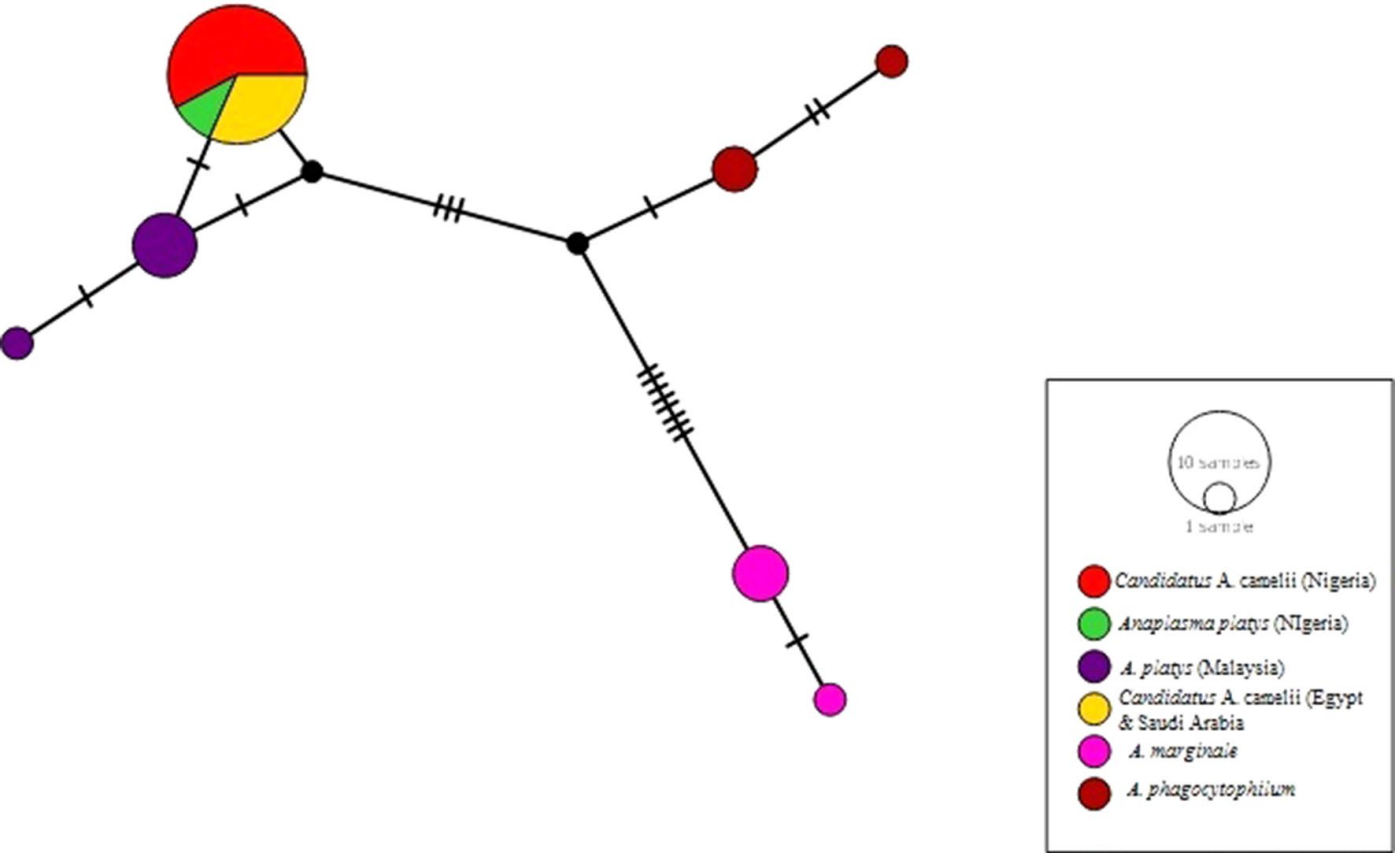
Median joining network of “*Candidatus* Anaplasma camelii” haplotypes based on *16S* rDNA sequences and its relatedness with other species of *Anaplasma*

Only one haplotype was found in this study (Fig. 3), which is similar to the haplotype detected from other “*Ca*. A. camelii” in Egypt and Saudi Arabia based on the sequences retrieved from the NCBI database. This haplotype differs slightly by a single mutation from *A. platys* of dogs in Malaysia (GenBank: KU500910). Furthermore, it also differs by 3 mutations from *A. phagocytophilium* and by 8 mutations from *A. marginale* (Fig. 3).

## Discussion

This study confirmed the occurrence of several tick-borne pathogens and the species diversity of ticks infesting camels in Nigeria. The overall rate of tick infestation in this study was 52.3%, lower than 80.0% reported by Abdullahi et al. [47] in Kebbi state, Nigeria. Differences between the two studies could be basically attributed to the smaller sample size in the latter study. Other factors possibly attributing to differences in tick infestations include geographical distribution, climatic factors, the management system as well as the frequency of acaricides application. We observed high numbers of male ticks compared with female ticks which is unsurprising considering the fact that the latter are known to detach from their host few days after feeding to oviposit, while the males stay longer for weeks before detaching [48].

In this study, ticks from Nigeria were morphologically identified and confirmed using molecular markers targeting several genes. So far, studies on tick identification in camels from Nigeria were based on morphology [6, 47, 49, 50]. Combining several mitochondrial markers (*12S*, *16S* and *cox*1), we were able to identify several species of *Hyalomma* ticks. The use of these markers has been increasingly useful for tick identification in several studies [51–54].

*Hyalomma dromedarii* was the most frequent tick species encountered in this study. Similar observations have also been reported by other researchers on ticks of camels in Nigeria [47, 49, 50], within other African countries [55, 56] and other parts of the world [57, 58]. The dromedary camel was found to be the preferred host for this tick species, but it also infests sheep, goats, cattle and horses [29]. In another study conducted at a single site in Nigeria, Kamani et al. [6], reported *H. impeltatum* as the most prevalent tick species of camel. We attributed some reasons for these differences. First, we sampled three study locations in north-western Nigeria. Secondly, we sampled towards the end of rainy season while Kamani et al. [6] sampled throughout the dry season. Nonetheless, previous studies on tick abundance of camels and the influence of season in both Kano and Sokoto states reported *H. dromedarii* as the most prevalent tick species of dromedary which was not influenced by season as this tick species showed preponderance on camels during both dry and wet seasons [50, 59]. Most likely, abiotic factors such as temperature and humidity may play a role in this observation, but this remains highly speculative.

*Hyalomma impressum* was the least prevalent tick species with only two specimens collected. This corroborates the observation of previous studies on ticks of camels in Nigeria [6] and Algeria [60] where two and three specimens respectively were collected. It is likely that environment as well as sampling time could also impact the prevalence of *H. impressum.* All other species of the genus *Hyalomma* such as *H. rufipes*, *H. truncatum* and *H. impeltatum* as well as *A. variegatum* and *R. evertsi evertsi* have been reported infesting camels in Nigeria [6, 47, 49].

*Rickettsia aeschlimannii* belongs to the spotted fever group of *Rickettsia* and is maintained and/or transmitted primarily by ticks. The MIR of *R. aeschlimannii* in ticks collected from camels was 7.8–11.5% across the three study locations (Kano, Jigawa and Sokoto states) and 5.6–36.8% across tick species. This confirms the presence of this species of *Rickettsia* in ticks infesting camels in Nigeria. A previous study in Kano, Nigeria reported an infection rate of 23.8% in ticks from camels [6]. The prevalence of pathogens detected in ticks is shown here as the minimum infection rate, assuming that only one sample is positive in each positive pool. Of course, this approach is only approximate and prevalence rates for the identified pathogens might be higher than reported in the present study.

All isolates in this study were identified as *R. aeschlimannii*, suggesting that the organism is endemic and widespread among *Hyalomma* tick species infesting camels in Nigeria. Furthermore, this bacterium has been detected in several countries within Africa in *Hyalomma* ticks [60–62]. The highest prevalence of *R. aeschlimannii* DNA was detected in *H. rufipes*, which agrees with the report of previous studies in Nigeria [6], Egypt [62] and Senegal [61]. Furthermore, all species of *Hyalomma* ticks were positive for *R. aeschlimannii* DNA except *H. impressum*. Similar findings were registered in previous studies in Algeria and Nigeria [4, 6, 60].

Piroplasms of the genera *Babesia* and *Theileria* are tick-borne pathogens of livestock including camels. The overall MIR of piroplasms (*Babesia* spp.) in ticks from this study was low in addition to the non-detection of these protozoan parasites in the blood. This corroborates with previous reports on piroplasms (both *Babesia* spp. and *Theileria* spp.) of camels [63, 64]. Previously, a low prevalence of *Theileria ovis* was reported in blood of camels from Sokoto, Nigeria using reverse line blot (RLB) [63] and in *H. dromedarii* ticks in Saudi Arabia [64]. Nevertheless, a high prevalence of 74.5% has been registered in the blood of camels in Sudan [65].

In the present study, *B. occultans* DNA was amplified and confirmed by sequencing in *Hyalomma* ticks (*H. impeltatum* and *H. dromedarii*) for the first time after its first morphological description in the haemolymph of *Hyalomma* ticks over three decades ago in Nigeria [66]. The DNA of *B. occultans* has been detected in other species of ticks such as *H. asiaticum* in China [67], *H. marginatum* in Tunisia [68] and *Rhipicephalus turanicus* and *H. marginatum rufipes* in Turkey [69]. In addition, DNA of this pathogen has also been detected in the blood of cows in Italy displaying fever, anemia, and hematological alterations [20]. Furthermore, DNA of *B. caballi* was amplified in a *H. dromedarii* tick. The detection of *B. caballi* in our study may not be surprising considering the fact that both camels and horses are infested with similar tick species [70]. Previous studies on camel piroplasms have detected *B. caballi* in the blood of camels in Sudan [71], Jordan [70] and Iraq [72].

The low infection rate of *C. burnetii* in *Hyalomma* tick species reported in the present study is comparable with that reported elsewhere for *Hyalomma* ticks [53, 73]. Most of the positives were detected in *H. dromedarii* and only one in a *H. truncatum* tick (1.1%). In a similar study in Egypt, *C. burnetii* was detected in *H. dromedarii* exclusively [53], while in China, most of the infection was in *H*. *asiaticum asiaticum* [73]. Furthermore, while *Coxiella*-like bacteria have been found in ticks as endosymbionts and play a role in tick fitness, *C. burnetii* is responsible for Q fever in vertebrates including humans [3]. Since ticks serve as a carrier of *C. burnetii* in livestock, the close association between man and livestock could probably lead to human infections [74]. An epidemiological survey among veterinarians and other high-risk individuals with regular contact with animals showed a high antibody titre to *C. burnetii*, suggesting possible transmission [75, 76].

Anaplasmosis in camels due to *Anaplasma marginale* causes subclinical disease as registered in other studies [77, 78]. In our study, camels from the three study areas tested positives to a novel species of *Anaplasma* named “*Ca*. A. camelii” by Bastos et al. [79]. This species is genetically related to *A. platys* [10, 12, 63, 79]. An earlier study in one of the study areas (Sokoto) reported a high prevalence for *A. platys* in camels [63]. The overall prevalence of “*Ca*. A. camelii” in camels in our study was 40.3%, which is comparable to results reported by Lbacha et al. [13] in Morocco, but higher compared with data from China (7.20%) [10] and Tunisia (17.70%) [12]. The variations in prevalence rates may result from differences in husbandry practices, tick control programmes and reservoir hosts [12].

Phylogenetic analyses in our study based on DNA sequencing clusters the *A. platys* reported earlier in one of the study areas (Sokoto state) (GenBank: KJ832066-KJ832067) with 99.5% identity to that obtained in our study (GenBank: MN396629-MN396638). It is therefore possible that the *A. platys* as earlier reported by Lorusso et al. [63], could be “*Ca*. A. camelii”. The *16S* sequences generated in the present study were identical to other “*Ca*. A. camelii” isolated in Saudi Arabia [79]. The haplotype analysis in our study shows that only one nucleotide differentiates *A. platys* with “*Ca*. A. camelii”, an observation similar to that observed by Sazmand et al. [14] in Iran.

Risk factors associated with “*Ca*. A. camelii” infection indicate that the female camels were more infected compared with the males corroborating previous studies [12, 63]. Immunosuppression associated with pregnancy and lactation has been attributed to be responsible for this observation [80]. The opposite was the case with respect to tick infestation, as more male camels were infested compared with females. It could also be that the male camels despite being more infested with ticks due to their natural behavior for space triggered by androgenic hormones, had better immunity against tick-borne infection than the females. A poor body condition score was a risk factor to both tick infestation and infection with “*Ca*. A. camelii” infection.

Older animals were more often infested with ticks as well as positive for “*Ca*. A. camelii” DNA than younger camels. According to Azmat et al. [81], the infection rate of camels with anaplasmosis increases with age. The occurrence of “*Ca*. A. camelii” infection in our study was positively associated with the presence of ticks. This finding confirms previous reports on anaplasmosis of camels [81, 82]. Also, *A. marginale* is responsible for bovine intra-erythrocytic anaplasmosis in bovines, but we did not find *A. marginale* in the investigated camels and ticks, an observation that has also been reported by other researchers [10, 11]. Furthermore, it has been postulated that dromedaries are not relevant reservoirs for already named *Anaplasma* species which include *A. marginale*, *A. centrale*, *A. phagocytophilum* and *A. bovis* [12].

## Conclusions

This study revealed the occurrence of different tick species and different tick-borne pathogens in ticks infesting camels as well as in their blood in Nigeria. We identified several subspecies of *Hyalomma* ticks and their associated tick-borne pathogens. Pathogen DNA detected in ticks using PCR and sequencing includes *R. aeschlimannii*, *B. caballi*, *Babesia* spp. and *C. burnetii*. Furthermore, we amplified *B. occultans* DNA in *Hyalomma* ticks infesting camels in Nigeria. “*Candidatus* A. camelii”, a novel species variant of *Anaplasma*, was the only pathogen amplified in the blood of the investigated camels. The detection of the two zoonotic pathogens, *R. aeschlimannii* and *C. burnetii*, may necessitate further investigation on the role of camels in their maintenance and reservoir status.

## Data Availability

All data generated or analyzed during this study are included in this published article. All newly generated sequences were submitted to the GenBank database.

## Abbreviations

MIR: minimum infection rates
TBP: tick-borne pathogens
BLAST: Basic Local Alignment Search Tool
PCR: polymerase chain reaction
CI: confdence interval
HM: *Hyalomma marginatum*
HR: *Hyalomma rufipes*
*cox*1: cytochrome *c* oxidase subunit 1
*glt*: citrate synthase
*omp*: outer membrane protein
